# The Value of Public Inquiries, Ethical Accountability, and Patient Voices: Reflections on the Infected Blood Inquiry

**DOI:** 10.1111/hae.70296

**Published:** 2026-04-20

**Authors:** Richard Gorman, Clive Smith, Bobbie Farsides, Emma Cave

**Affiliations:** ^1^ Department of Clinical and Experimental Medicine Brighton and Sussex Medical School University of Sussex Brighton UK; ^2^ The Haemophilia Society London UK; ^3^ The Infected Blood Memorial Committee London UK; ^4^ Durham Law School Durham University Durham UK; ^5^ Co‐convener of the Medical Ethics Expert Report for the Infected Blood Inquiry London UK

**Keywords:** ethics, haemophilia, infected blood inquiry, informed consent, public inquiries

## Abstract

**Introduction:**

This article contributes to the continuing conversation in *Haemophilia* about the UK Infected Blood Inquiry (IBI). Discussion within the journal to date has largely foregrounded professional and technical perspectives.

**Aim:**

This article aims to bring back into view two elements central to the Inquiry—patient voice and the roles of law and ethics—and consider what this means for how the lessons of the IBI are interpreted in clinical and public discourse.

**Methods:**

The article engages with published reflections and commentary to consider how responsibility has been framed, how consent has been understood, and what is at stake in the shaping of professional memory.

**Results:**

The article shows how narrowed framings of consent, appeals to clinical authority, and selective uses of language risk marginalising those infected and affected. It argues that such narratives continue to shape how responsibility is allocated and how the Inquiry will be remembered within professional discourse.

**Conclusion:**

Accountability, transparency, and recognition must remain central as the implications of the Inquiry continue to unfold, and as processes of reconciliation begin.

## 1 Introduction

In April 2025, 11 months after the publication of the UK Infected Blood Inquiry's (IBI's) final report, this journal published a review article by Professor Christine A. Lee [1]. Lee is an eminent haematologist, a founding editor of *Haemophilia*, and has previously been awarded the World Federation of Hemophilia's International Lifetime Achievement Award. Lee's article proposes to present a reflective perspective from the professional clinical community directly affected by the Inquiry's findings and expresses deep concern about how the Inquiry handled topics of complexity [1]. In doing so, the article runs the risk of casting doubt on the foundations of the Inquiry's conclusions.

The publication of the IBI Report represents a landmark endeavour in confronting historic failings in UK healthcare [2]. Its findings—including that over 3,000 people died due to the clinical use of infected blood products—have profound implications for medical accountability, regulatory oversight, and the foregrounding of patient voices in shaping healthcare policy, advancing justice, and embedding lived experience within institutional memory. In 2024, the UK Government published its response to the Report in which it accepted the vast majority of the recommendations in full or accepted them in principle: [3]

“The findings of the Infected Blood Inquiry are horrifying. A catalogue of failures at systematic, collective and individual level, contributing to more than 3,000 deaths that are attributable to infected blood, blood products and tissue. The report finds that these were largely avoidable.” ‐ Ministerial Foreword to the UK Government Response to the Infected Blood Inquiry [3]

The IBI's widely lauded efforts to confront historical harm in healthcare must not be diluted or diminished. As such, we believe that a robust response is required—one that foregrounds ethical analysis, reasserts the importance of patient narratives, and critically examines the assumptions underpinning the account presented. Without doing so, there is a real risk that future readers of Lee's reflection might conclude that the Inquiry was misguided, unfairly critical, or failed to appropriately attribute responsibility. Given its publication in *Haemophilia*, a journal with longstanding influence in the bleeding disorders field, these risks carry a unique weight and underscore the importance of engaging critically with its findings; the narratives that circulate in such forums shape professional memory. This commentary was the first article published in *Haemophilia* to discuss the aftermath of the Inquiry, and has since prompted several other responses [4, 5, 6]. We aim to further add to this conversation, as we believe it is important to guard against any misrepresentation and ensure the Inquiry's profound ethical and institutional significance is fully recognised and accounted for. In doing so, we acknowledge that haemophilia, if untreated or inadequately managed, carries substantial risk of serious bleeding, long‐term disability, and potential mortality. Our analysis situates the IBI's critique within the context of these clinical realities while focusing on the ethical and systemic dimensions of how historic decisions were navigated—and importantly what is at stake in how they are represented and remembered.

## 2 The Value of Public Inquiries

Lee's criticisms of the Inquiry are aimed at public inquires in general, inquiries into medical issues, and matters specific to the IBI [1]. We briefly consider the first two in this section before turning to her more specific concerns.

Statutory public inquiries are established by government ministers and conducted by an independent body to examine matters of significant public concern [7]. Public inquiries aim to find out what happened, what can be done, and how it can be prevented from happening again. Whilst a full defence lies beyond the scope of this reply, we note the significant body of evidence demonstrating the valuable legislative and policy changes that have resulted from them []. In this case, the IBI was established only after decades of campaigning by those infected and affected—many of whom have since died without recognition of wrongdoing or redress.

Lee is critical of the financial cost and length of the Inquiry [1]. It is true that inquiries addressing complex issues and reviewing substantial bodies of evidence to establish the truth necessarily require significant time and resources. Section 17(3) of the Inquiries Act 2005, however, expressly requires the avoidance of unnecessary expense. Moreover, the costs must be balanced against the benefits of uncovering the truth, preventing future mistakes and ensuring public access to proceedings.

Some inquiries, including this one, recommend financial redress where wrongs have been identified. Discussion of compensation in the context of the IBI requires particular care and sensitivity. Lee's article pays little attention to the ways in which people with bleeding disorders and their families have borne heavy costs, costs which will never disappear, but could be ameliorated by forms of recognitional justice, acknowledgment, and apology. There are references to the potential scale of compensation payments, which stemmed from a separate government commissioned report chaired by Sir Robert Francis KC [9]. The implication that recognition and accountability should be set against their economic implications is ethically uncomfortable when contextualised alongside the depth and duration of harm. It also overlooks that financial redress, however imperfect, is one of the few tangible mechanisms available to acknowledge these moral costs. Furthermore, the disaster itself was exacerbated by cost considerations—a reminder that economic reasoning has already imposed profound harm on this community [10]. Finally, if we are to reflect on costs, then it is worth critically reflecting on the costs borne by society in not achieving a publicly acceptable resolution, particularly in the loss of trust in healthcare systems and government.

We acknowledge the difficulty faced by clinicians who are required by law to give evidence to a public inquiry. Giving evidence can be a challenging and often stressful experience, particularly where professional judgement or practice is under scrutiny. However, contrary to Lee's view that the process was adversarial[1], inquiries are in fact inquisitorial in nature [7]. The IBI was concerned with exposing structural harms—scrutinising the roles of clinicians, regulatory bodies, and government alike. Inquiries can and do make findings that are critical of individuals or organisations where the evidence supports it, but they cannot impose sanctions or declare criminal culpability or civil liability.

Some of Lee's criticisms are pertinent to other public inquiries into medical issues. Lee frames the IBI as a ‘collision’ between government, medicine, and law. Yet medicine is always entwined with legal and governmental structures. Clinical practice does not take place in isolation; it is continuously shaped by regulation, policy, and accountability systems. From its inception, therefore, the IBI was about understanding systemic failures and restoring ethical accountability and trust with a harmed community.

Lee also claims that the Inquiry was ill‐equipped to understand the complexity of clinical practice [1], but in addition to the wealth of written and oral evidence reviewed, the Inquiry established numerous expert groups—including those on Medical Ethics, Public Health and Administration, Psychosocial Impact, Statistics, Clinical Practice, Bleeding Disorders, and Health Economics—to ensure a breadth and depth of expertise informed its work. Far from lacking the competence to assess past practice, the Inquiry embedded expert insight across domains, thereby strengthening its capacity to evaluate complex, systemic harms and address questions of institutional and ethical accountability.

## 3 Clinical Ethics, Risk, and Moral Responsibility

While recognising the historical context of medical practice is essential, the Inquiry also grounded its analysis in enduring ethical and moral principles such as respect for autonomy and non‐maleficence [11]. These principles were directly relevant to the practices under scrutiny, particularly around consent. This is thus not merely a matter of how individuals acted, but of what healthcare cultures and systems permitted and normalised. The article's defence of historical norms around consent implies that contemporary ethical standards are being unfairly applied in retrospect. This misrepresents both the Inquiry's findings and the ethical significance of consent at the time.^1^ The Nuremberg Code established informed consent as fundamental to medical research in 1947. The Medical Ethics Expert Report to the IBI discusses the temptation of this kind of historical revisionism in detail, noting “*although people may have been operating in line with contemporary moral norms, their actions can be challenged where we can identify relevant fundamental moral values which should have been respected, irrespective of time or place” (p.7)* [13]. The claim by Lee that ‘formal written consent was not required’ [1] reflects a procedural defence that sidesteps the central ethical issue of whether patients and families were meaningfully informed and able to exercise choice. Characterising consent in such a procedural way overlooks the substantive ethical failures—of disclosure, dialogue, and autonomy—that the Inquiry uncovered. Ultimately, this is not simply a question of judging the past by today's standards, but of recognising that ethical concerns were present and raised—though rarely leading to action.

Lee asserts that clinicians made ‘an enormous contribution’ to enabling ‘a good quality and expectancy of life’ in people with haemophilia [1]. Yet we must recognise that good intentions and acts can coexist with failures of care. The IBI does not condemn all practice, but aspects of it. As such, these achievements must be considered alongside the devastating, preventable infections that profoundly affected so many. Without this recognition, there is a risk that patients’ suffering will appear as little more than collateral damage in the pursuit of scientific progress. The celebratory tone toward researchers who first examined the ‘quality of life’ of boys with haemophilia is uncomfortably disconnected from reflection on how infected blood products would later devastate the quality of life of that very same group [1]. The commentary's defence that clinicians were simply delivering ‘best practice’ leans heavily on the intentions and innovation of a select group of clinicians, while minimising the consequences of their decisions [1]. Clinical good intentions alone do not constitute ethical responsibility. Benefit must always be assessed in the context of the risks of harm, respecting the principle of non‐maleficence in balance with a desire for beneficence [11].

As Walker argues, moral practices ‘cannot be extricated from other social practices, nor moral identities from social roles and institutions’ (p.17) [14]. This complicates Lee's reliance on professional experience and standing, given how institutional roles can constrain moral reflection, especially where cultures of expertise discourage dissent or prioritise innovation. Indeed, in their reading of the Inquiry, McKee draws attention to how one clinician, unconvinced by emerging evidence about blood‐borne transmission, came to overly influence policy through simultaneous membership of multiple advisory bodies—including patient‐facing organisations [15]. This context challenges Lee's defence of clinical reasoning at the time, and raises difficult questions about whose voices were heard, amplified, or excluded. By stating that the issues under scrutiny in the Inquiry were too complex for lawyers, the commentary reinforces a hierarchy of knowledge that privileges medical authority while marginalising legal, interdisciplinary, and patient perspectives. Bishop James Jones, who chaired the Hillsborough Independent Panel into the deaths of 97 football fans at an FA Cup semi‐final, titled his report on the family's experience, “The Patronising Disposition of Unaccountable Power” [16]. Lee's framing of the Inquiry lives up to this epithet. Indeed, this discursive approach treats other ways of knowing as less valid or true—reinforcing what medical sociologists have long identified as the epistemic dominance of clinical expertise [17]. Such an appeal to clinical expertise and virtue also fails to account for how cultures of medical paternalism have shaped clinical encounters in ways that have historically limited patient agency [18].

Lee's commentary rightly highlights the importance of trials that established the efficacy of prophylaxis and secured its funding [1]. However, in celebrating this progress, she overlooks the profound human cost that accompanied it. Prophylaxis was undoubtedly valuable for the haemophilia community, yet the ethical failure lay not in the rationale or frequency of treatment, but in the manner of its implementation. There is a risk, that in celebrating clinical progress this depiction obscures a core ethical concern: that many people were placed on high‐risk treatment protocols without informed consent and suffered life‐limiting consequences as a result. Indeed, the commentary's presentation of high‐risk treatment decisions as always unavoidable alternatives to mortality oversimplifies the complex ethical terrain of exposing haemophilia patients to serious risks during a period of medical uncertainty. For example, the IBI heard from people with mild bleeding disorders who had been treated with blood products for minor surgery, with one recalling they *“would never have consented to this form of treatment, unless of course it was a life saving necessity which of course it was not”* (emphasis original, IBI Report, volume 2, p.7–8) [2]. This is not to say that treatment did not have value—we can agree that treatment avoided much harm—however, we must recognise that deliberations on cost‐benefit are complex, and the ethics of such should not be flattened. Other recent reflections have built on Lee's commentary, suggesting that treating bleeding events inadequately could have represented an even greater moral failure [4]. Yet this framing again evades a central concern: that patients and families were not empowered to share in these harm–benefit calculations. Even where clinical options are severely constrained, informed consent does not become redundant. Ethical responsibility requires not only balancing potential harms and benefits but also ensuring those affected are fully informed and able to participate in decisions about their care.

## 4 Language Matters: Knowledge Hierarchies and the Power to Define

Lee objects to the Inquiry's use of the term ‘experimented’ to describe the involvement of four children within a particular study [1]. She instead describes the study as retrospective, claiming that no formal recruitment occurred and that the participants were only identified later through existing samples and data. This is a form of boundary work, a discursive strategy that seeks to protect clinical practice from ethical scrutiny by maintaining distinctions between research and treatment. Yet the concern does not rest on whether data analysis was retrospective. It rests on the fact that children were placed on risky regimens without adequate disclosure or consent, and in contexts where their biology was used to generate knowledge (raising, as in the case of Henrietta Lacks, additional ethical concerns about unconsented use and lack of recognition in biomedical research) [19]. However these practices are labelled, they bear the ethical characteristics of experimentation. Ultimately, whether we linguistically label this experimentation or not, the ethical failure remains the same: patients and their families were not given meaningful information or choices. Serious illness makes openness and shared decision‐making more, not less, essential.

Where patients or parents were not told about risks or treatments, or were not given meaningful choices, their autonomy was compromised. As Makris and O'Mahony observe, many clinicians came to see hepatitis as a ‘small price to pay’ for more convenient treatment [20]. This paternalistic normalisation of harm raises serious concerns: whether patients were adequately informed, whether risks were downplayed, and whether meaningful choices were ever really possible. Lee describes how retrospective analysis of infected haemophilia patients helped inform early strategies for managing HIV beyond the haemophilia community [1]. But this discursive move threatens to imply that the harms endured by this community are redeemed by their scientific utility. It veers towards a utilitarian and sacrificial logic, in which the suffering of one group is justified by the benefits it enables for others.

While the commentary suggests that disclosures were made in supportive settings, such as with a family therapist present [1], this was not the experience of most affected families [2, 24]. Indeed, this is contrary to direct evidence received by the Inquiry from one core‐participant and campaigner, Mark Ward. Mark, then a patient at the Royal Free Hospital (where Lee bases these recollections), described the harrowing moment his HIV test result was shouted across the haemophilia centre's waiting room to him and his family, telling him he was HIV positive [25]. Similarly, the Expert Report to the IBI: Psychosocial Issues discusses a witness report describing: *“how a haematologist treating her 2‐year old son with haemophilia “came down a corridor and told us that he (her son) had HIV. He didn't even take us into a room, he just told us in a corridor in front of other patients”*.” (p.12) [24] The inquiry, sadly, heard of many comparable experiences. Thus, while this may reflect the author's own memory of her Haemophilia Centre's practice, such retrospective anecdote may overstate its representativeness and should not be taken as exemplary of practice elsewhere and at large. Presented without qualification, such accounts can inadvertently recast exceptions as norms, softening the ethical scrutiny warranted by the broader body of evidence. The Inquiry heard extensive evidence of distressing, unsupported, and in some cases callous communication of life‐changing diagnoses with little or no psychological support [24]. Other accounts have emphasised gratitude for the care received and respect for the personal dedication of particular clinicians and these experiences should of course not be dismissed [5]. Indeed, the ethical force of the Inquiry lies precisely in recognising this plurality of experience—that gratitude for individual care can simultaneously coexist with, but does not erase, the reality of systemic failure and enduring harm.

Lee refers to patients being ‘unknowingly infected with HIV’ [1], language that diminishes institutional accountability and can operate as symbolically violent—a form of social power in which linguistic framing obscures systemic responsibility and normalises harm [21]. The issue is how the phrasing directs attention, not the epistemic circumstances themselves: it invites the reader to treat harm as mischance, rather than the outcome of organisational choices and tolerances for risk. By the early 1980s, transfusion‐transmitted AIDS was an emerging concern (and concerns about transfusion‐hepatis emerged even earlier) [2, 22, 23]. However, the issue goes beyond simply what was known, to what was done—and ultimately from an ethical perspective, whether patients were adequately informed or protected. Overly centring the ‘unknown’ in this discourse obscures the hierarchies of knowledge, trust, and responsibility that shaped clinical decisions—and limited patients’ ability to question or refuse them.

Whilst discussing the development of prophylaxis, Lee criticises the Inquiry for failing to consider international practice, noting prophylaxis regimes were used in bleeding disorders care in Europe and North America [1]. However, this risks oversimplifying important distinctions and obscuring the specific conditions under which UK patients were exposed to high‐risk treatments. The relevant question is not whether prophylaxis was used elsewhere, but *how* it was implemented—particularly in relation to clinical oversight, informed consent, autonomous decision‐making, and the sourcing of blood‐products. At Treloar's, for example, the IBI notes a concerning trend, where *“it became a policy to encourage prophylaxis even where pupils themselves would have preferred not to have it.”* (Volume 3, p.43) [2]. Certainly, international practice—and perhaps more significantly international response—varied considerably, and Lee omits comparative discussion of where other countries took earlier precautionary steps. Indeed, nor is any mention made of the fact that Ireland, Canada and the US paid extensive compensation to patients as a result of the regimes employed [26]. Ultimately though, the Inquiry's concern was not the existence of prophylaxis abroad, but whether appropriate steps were taken in the UK to reduce foreseeable harms. The Inquiry notes that opportunities were missed to *“respond to serious risks of infection by making adjustments to treatment regimes to make them safer”* (Volume 1, p.4) [2]. Ethical action under uncertainty demands communication, transparency, precaution, and responsiveness—and above all, an obligation to involve patients meaningfully in decisions. The issue is not whether the full scale of risk was known, but whether sufficient concern existed to justify greater caution, disclosure, or change. Lee's commentary itself notes studies from WWII that suggested a caution around pooled plasma [1]. Indeed, Lee herself recalls writing that she had a ‘nasty feeling that NHS concentrate is going to turn out safer’ but does not reference whether this uncertainty led to an opportunity to pivot practice [1]. It is not within the scope of this piece to revisit the Inquiry's extensive exploration of what was, or should have been, known about the risks of hepatitis and HIV at the time of the events under examination (detailed and documented in Volume 3 of the Inquiry report) [2]. However, we would simply point towards its synthesis of evidence that concludes:

“There can be little doubt that possibly by March 1982, and certainly from July 1982 onward, it was known in the UK to both some clinicians and some within government that there was a real risk that blood, and blood products in particular, might transmit the cause of AIDS.” ‐ Infected Blood Inquiry Report, volume 1, p.25 [2].

Furthering its defensive stance, Lee's commentary quotes an article from 1986 stating that ‘effective treatment is rarely free from adverse effects’ [1]. The inclusion of this within a commentary on Infected Blood acts to diminish the scale of the harms examined by the Inquiry. The side effects under discussion were not routine complications, but the transmission of HIV and hepatitis to thousands of people—outcomes with profound and often fatal consequences. Presenting these as simply the adverse effects of effective treatment blurs the line between the inherent risks of medical advancement and the ethical failings of systemic inaction, inadequate transparency, and absent consent. In writing about the Inquiry, McKee highlights the long‐standing use of rehearsed institutional lines, such as invoking claims about the ‘best treatment available’ or portraying infections as ‘acceptable side effects’—that the Inquiry found to be misleading and ethically evasive [15]. Lee's uncritical return to similar framings threatens to perpetuate that same institutional evasiveness. The commentary's description of deaths caused by ‘the recklessness and obstinacy of officialdom’ [1] further reinforces this tendency to deflect accountability solely onto faceless and far‐removed bureaucratic actors whilst overlooking how other failures were perpetuated, institutionally and clinically.

## 5 Missing Voices, Ethical Absence

The commentary offers insights into the experiences of senior healthcare professionals who navigated haemophilia care during a period of dramatic clinical, technological, and social change, and whose voices—the commentary argues—are largely absent from both the Report and public and professional discourse about Infected Blood [1]. Whilst it is true that some clinicians were unable to give evidence, the Inquiry did take substantial evidence from clinicians, including from the commentary's author themselves [2]. While the passage of time inevitably meant that some clinicians were unable to give evidence due to illness or death, to forgo the Inquiry on that basis would only have compounded the injustice to the affected and infected, leaving them to endure ongoing harms without acknowledgement, accountability, or the prospect of redress. Likewise, any ‘absence’ of clinical voices, we would respond, must be understood alongside the recognition of the many patients who equally did not survive to testify, whose stories remain unacknowledged, and whose silencing is in itself ethically significant.

Disappointingly, the voices of infected or affected individuals are not acknowledged or engaged within the commentary. This absence—of patient experience, of apology, of any direct expression of regret—is itself ethically meaningful. It represents what Foucault termed the medical gaze—a modality that renders patients visible only as clinical subjects, erasing their agency, narratives, and voice [27]. That some clinicians made a ‘major contribution’ to haemophilia care does not absolve the profession of the grave failures that occurred during the same period [1]. Both truths can—and must—be held simultaneously. A response to the Inquiry centred solely on professional legacy acts to reproduce the very patterns of marginalisation and defensiveness the Inquiry sought to redress. As Wherry notes of Infected Blood, ‘it is vital that clinicians working with people affected by this issue are mindful of the need for trauma aware care to prevent further harm’ (p.26) [28]. This reminds us that recognition alone is not enough; clinical and institutional actors must take responsibility for repairing harm and the instigation of processes of reconciliation. Yet Lee's commentary falls short, glossing over the enduring damage by prioritising professional perspectives at the expense of patient voices and the ethical imperative for accountability and justice.

## 6 Innovation, Responsibility, and the Lessons of Infected Blood

Lee's article concludes by arguing that ‘scientific enquiry of innovative treatments is essential and there should not be victimisation if unforeseen side effects occur’ [1]. The use of the word “unforeseen” completely overlooks the Chair's emphatic findings on page 1 of the Inquiry Report in which he states that much of the disaster could have—and indeed, should have—been avoided [2]. Describing harm as merely the unfortunate cost of innovation actively perpetuates the institutional defensiveness and patterns of silence that necessitated the Inquiry in the first place. A continued focus on ‘unforeseen’ would seem to minimise reflection on the harm committed, deflecting systemic and institutional responsibility, and perpetuating a clinician‐centred narrative. As the Inquiry documents in detail, many clinicians remained reluctant to change treatment practices [20]. The harm was not unforeseeable; it was foreseen and tolerated. The IBI is clear that the extent of the disaster was not inevitable and the response should have been better. To quote the Inquiry report:

“What occurred was not an inevitable course of events. It was not a tragic accident, in the sense of something that was unavoidable. It was not the result of an unknown against which steps could not be taken effectively.” – Infected Blood Inquiry Report, Volume 1, p.19. [2]

Innovation does not excuse the normalisation of risk. Indeed, rather than a normalisation of risk in the context of irreversible therapies as the commentary seems to advocate for, the IBI stands as a stark reminder of what is at stake when innovation outpaces ethical accountability. This is especially salient in present debates about advanced therapies such as gene therapy, where the need for transparency, patient agency, and robust safeguards is paramount [29, 30]. The historic ethical and systemic failings uncovered by the IBI provide important lessons that could guide the introduction and uptake of emerging gene and cell therapies. The IBI's findings emphasise the need for meaningful and informative discussions with patients, ensuring they are empowered to understand both known and potential unknown risks, and centring practices of shared decision‐making alongside joint efforts to foster trust and anticipate potential harms.

Building on Lee, others writing in this journal have suggested that the Inquiry itself risks undermining public confidence in science [4]. However, we would argue that this inverts the real danger, and that it is in fact the downplaying of the findings of the Inquiry that risks eroding patient and public trust in medicine. Framing scrutiny as a threat to science risks obscuring the Inquiry's central achievement in making visible systemic failures and harms that have long been denied. The Inquiry's conclusions are not an obstacle to innovation, but a reminder that innovation must proceed with transparency, accountability, and patient voice at its core. The lessons of Infected Blood must not be softened by appeals to professional legacy or reframed as the inevitable costs of progress. Anything less would risk repeating the very patterns of silence and harm that the Inquiry was established to confront. There have been calls to ‘let the dust settle’ [5], and certainly time will be needed for both families and professionals to process the Inquiry's findings, but what is also needed are processes of reconciliation and memorialisation in order to acknowledge past harms and begin rebuilding trust.

## Funding

The authors have nothing to report.

## Ethics Statement

Ethical approval was not required for this article, as it does not involve original research with human participants or animals.

## Conflicts of Interest

The authors declare no conflicts of interest

## Data Availability

Data sharing not applicable to this article as no datasets were generated or analysed during the current study.

## Endnotes

1 What does potentially risk becoming an anachronism though is the invocation of ‘evidence‐based medicine’ as a justification for historical clinical decisions – a phrase emerging in the early 1990s.^[^ ^12^ ^]^ Overextending an EBM framework here risks obscuring the role of local cultures of care in shaping bleeding disorders care at the time.
